# Disturbance type determines how connectivity shapes ecosystem resilience

**DOI:** 10.1038/s41598-021-80987-1

**Published:** 2021-01-13

**Authors:** Ryan M. Pearson, Thomas A. Schlacher, Kristin I. Jinks, Andrew D. Olds, Christopher J. Brown, Rod M. Connolly

**Affiliations:** 1 Australian Rivers Institute-Coast and Estuaries, School of Environment and Science, Griffith University, Gold Coast, QLD 4222 Australia; 2 School of Science and Engineering, University of the Sunshine Coast, Maroochydore, DC 4558 Australia

**Keywords:** Climate-change ecology, Ecological modelling, Ecological networks, Ecosystem ecology, Theoretical ecology, Ecology, Ecology, Environmental sciences

## Abstract

Connectivity is fundamentally important for shaping the resilience of complex human and natural networks when systems are disturbed. Ecosystem resilience is, in part, shaped by the spatial arrangement of habitats, the permeability and fluxes between them, the stabilising functions performed by organisms, their dispersal traits, and the interactions between functions and stressor types. Controlled investigations of the relationships between these phenomena under multiple stressors are sparse, possibly due to logistic and ethical difficulties associated with applying and controlling stressors at landscape scales. Here we show that grazing performance, a key ecosystem function, is linked to connectivity by manipulating the spatial configuration of habitats in microcosms impacted by multiple stressors. Greater connectivity enhanced ecosystem function and reduced variability in grazing performance in unperturbed systems. Improved functional performance was observed in better connected systems stressed by harvesting pressure and temperature rise, but this effect was notably reversed by the spread of disease. Connectivity has complex effects on ecological functions and resilience, and the nuances should be recognised more fully in ecosystem conservation.

## Introduction

Multiple stressors are affecting multiple ecosystems on Earth^1–6^. Ecosystem resilience describes the capacity for an ecosystem to maintain a stable state in the face of disturbance, either by resisting change or by rapidly recovering from disturbance effects, thus avoiding a regime shift to an alternative stable state^7^. How readily ecosystems resist, or recover, from the consequences of external pressures depends in part on the type of perturbations^8^, the responses of organisms within them, and the connectivity of habitat networks that surround them^7,9^. Connectivity also shapes how disturbance spreads through ecosystems and modifies the strength of ecological processes, affecting ecosystem resilience^10,11^.

Animals perform many ecological functions that can stabilise or maintain ecosystem structure and function when disturbed, but disturbances themselves can also reduce the efficacy of these mitigating functions. In fragmented habitats, for example, plant production may be limited when pollination declines, but the movement of animal pollinators between patches can counteract this^12^. External stressors may impede travel between plants resulting in declines in pollination rates^13^. This affects ecosystem resilience by reducing the reproductive capacity of certain plants, potentially triggering changes in the species composition and functional capacity of ecosystems^13^. Similarly, fishing on coral reefs can remove herbivorous fish that control algal growth via grazing e.g.^5,14,15^. Phase-shifts can occur when herbivory rates are reduced because coral settlement and expansion are inhibited by algal overgrowth^16,17^.

In spatial habitat mosaics, theory posits that the number and architecture of connections plays a role in constraining and shaping the way disturbance events modify ecological functions^18,19^. Yet, there is scant empirical data to test these predictions, especially how connectivity and disturbance types interact to control key ecological processes. We expect that highly connected networks will be more resilient to most individual stressors that affect only part of a system (e.g. harvesting near to protected areas). We refer to ‘part of a system’ in the respect that there be some capacity for unstressed areas to provide support to the stressed areas in resisting or recovering from disturbance. For animal-derived functions (e.g. pollination, grazing), the capacity for unaffected areas to provide this support will depend on the dispersal capacity and behaviour of the animals performing the function. For some animals, this may be many thousands of kilometres, while for others it may be metres. Thus, dispersal capacity should be considered when assessing connectivity and disturbance to ensure appropriate spatial scales are considered.

When external stressors impede the performance of animals in parts of the system, better-connected habitat networks will be less affected because animals can be re-supplied more readily via unperturbed alternative pathways e.g.^20–22^. By contrast, stressors like disease, which rely on interactions between organisms to spread, may have the opposite effect in better-connected systems e.g.^23^. Despite the clear benefits for ecosystem management that might come from understanding these dynamics, the nature of the connectivity-resilience relationship remains poorly understood. Here we aim to assess the role that connectivity plays in shaping animal functional responses to single and multiple disturbance events of different types. Specifically, whether the type of stressor(s) determines how connectivity shapes the resilience of ecosystems.

## Experimental networks

**Figure 1:**
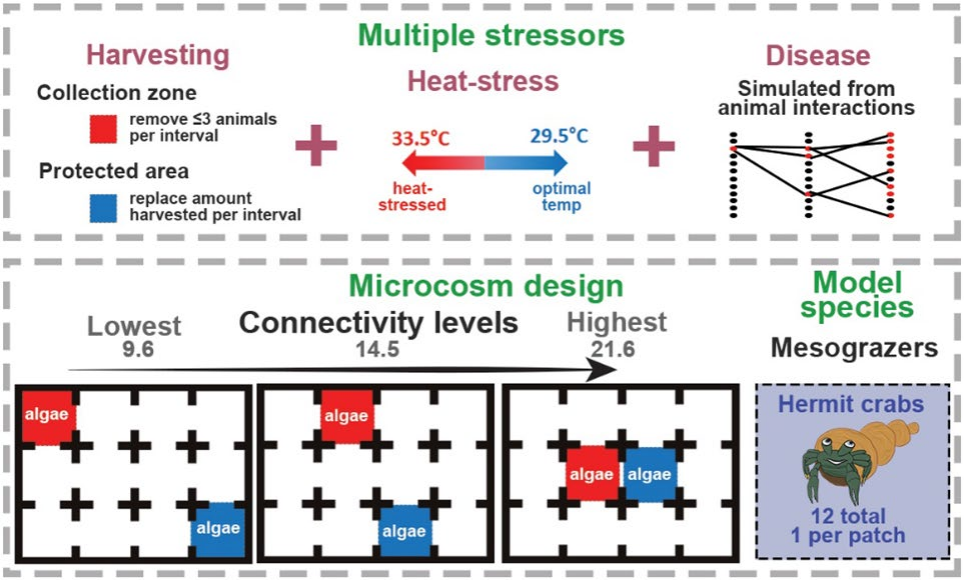
Experimental design with multiple stressors (top) and connectivity levels (bottom). Stressors were applied individually and in combination. Algal pellets were added into the coloured patches, with the amount consumed recorded at each interval.

We address these questions in experimental microcosms. Microcosms do not attempt to mimic the complexity of real-world ecosystems and instead provide capacity to precisely control multiple factors, allowing investigations into specific components of complex systems. As such, microcosm experiments such as ours provide insight into the nature of relationships between the components that are investigated, but additional experiments are needed to then validate real-world outcomes. We tested the relationship between connectivity and grazing performance (as a proxy for ecosystem resilience) under multiple stressors by measuring the removal of algal pellets by an invertebrate marine grazer, the yellow-footed hermit crab (*Clibanarius virescens),* at three connectivity levels exposed to several disturbance treatments (Fig. 1). *C. virescens* is an abundant grazer, widespread throughout the Indo-Pacific, and with known temperature sensitivities^24^. In our experimental system, we equate higher grazing rates to higher resilience because grazing is instrumental in influencing ecosystems resilience (e.g.^20,25^). Marine systems with higher grazing rates are likely to be more resilient in resisting a phase-shift towards algal dominance when disturbed^17^. Similar methodological approaches and inferences have been used in recent microcosm studies aimed at assessing the effect of different aspects of connectivity^26^ and temperature stress^24^ on the grazing performance of *C. virescens*.

We subjected 12 grazers per trial to three stressors (heat-stress, harvesting, disease), applying them individually and in combination to arenas with three levels of connectivity between habitat patches (Fig. 1). Connectivity was manipulated by altering the location of two habitat patches containing algal food to simulate three spatial scenarios of habitat mosaics in a landscape (Fig. 1). A modified measure of closeness provided a connectivity metric for each arena (Fig. 1). Physical disturbance agents (harvesting of grazers and heat-stress) were applied directly to a part of the arena while a third stressor (disease) was simulated from interactions observed on video recordings, followed by application of a consumption penalty to infected individuals proximal to food (Fig. 1).

## Methods

### Connectivity confers resilience

**Figure 2:**
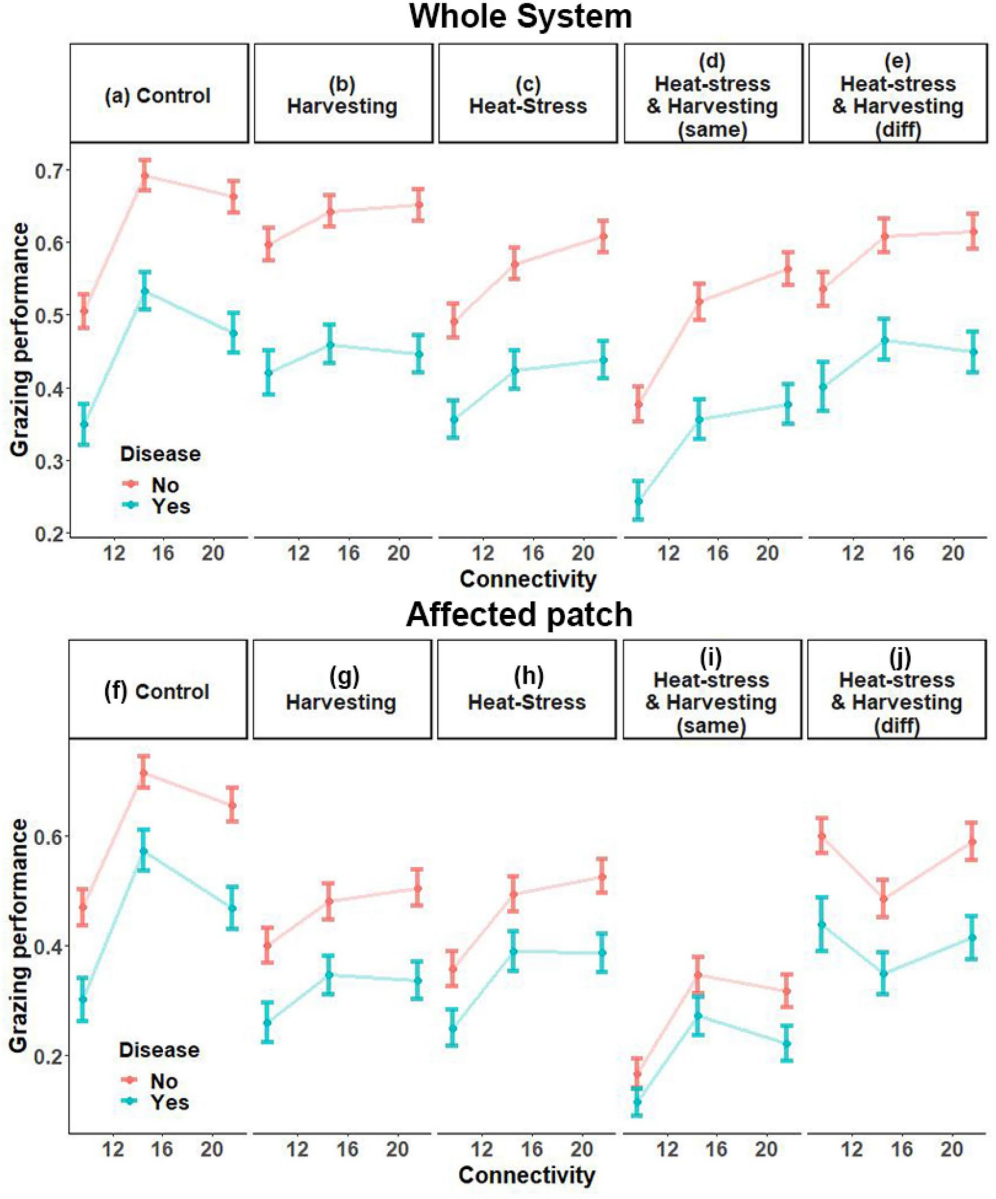
Grazing performance in response to ten experimental disturbance treatments at three connectivity levels. Grazing performance is algal consumption as a proportion of total algae available (mean ± SE). In d: both stressors were applied to the same patch (same). In e: stressors were applied to different patches (diff).

We observed inherently higher grazing performance in better-connected, unperturbed systems (Fig. 2a,f). The two better-connected systems (connectivity = 14.5; 21.6) had significantly higher grazing performance than the least-connected system (connectivity = 9.6), demonstrating a positive, non-linear, relationship between connectivity and ecosystem function in the controls (Fig. 2a,f; S1, S2). Physical disturbances, however, did not appear to dramatically alter this relationship at the system level (Fig. 2b,c), despite clear impacts on performance in the affected patch (Fig. 2g,h).

Each disturbance regime resulted in a significant decrease in grazing performance within the affected patch (Fig. 2f:j; S2), but not always across the entire system (e.g. Fig. 2a:e; S1). Impacts within affected areas were sometimes offset at the whole system level by increases within unstressed patches because the stressors themselves triggered animal aggregations in unstressed regions. For example, the animals typically left heat-stressed areas, sometimes finding refuge in the unstressed patch. This meant that unstressed protected areas sometimes returned higher grazing performance than in the control scenarios, offsetting losses in the affected patch and resulting in no significant change in performance across the whole system (Fig. 2b,c).

### Multiple stressor scenarios

Multiple stressors had variable effects on the shape of the connectivity-resilience relationship (Fig. 2; Extended data Fig. 2). When heat-stress and harvesting were applied to the same patch, grazing performance was heavily reduced under all connectivity scenarios, but the shape of the connectivity-resilience relationship remained positive (Fig. 2d,i). However, when applied to different patches, these effects were strongly antagonistic. Heat-stress offset the effect of harvesting, creating a slight negative relationship between connectivity and resilience. There was a loss in grazing performance at higher connectivity levels and, by contrast, a slight gain at the lowest connectivity (Fig. 2e,j). Heat-stress likely encouraged animals to move away from the hotter patch, into the harvesting patch. These patches were in closer proximity in better-connected systems, making animal congregations in the harvesting patch more likely, thus increasing the relative impact of harvesting and changing the shape of the connectivity-resilience relationship.

### Disease interactions

**Figure 3:**
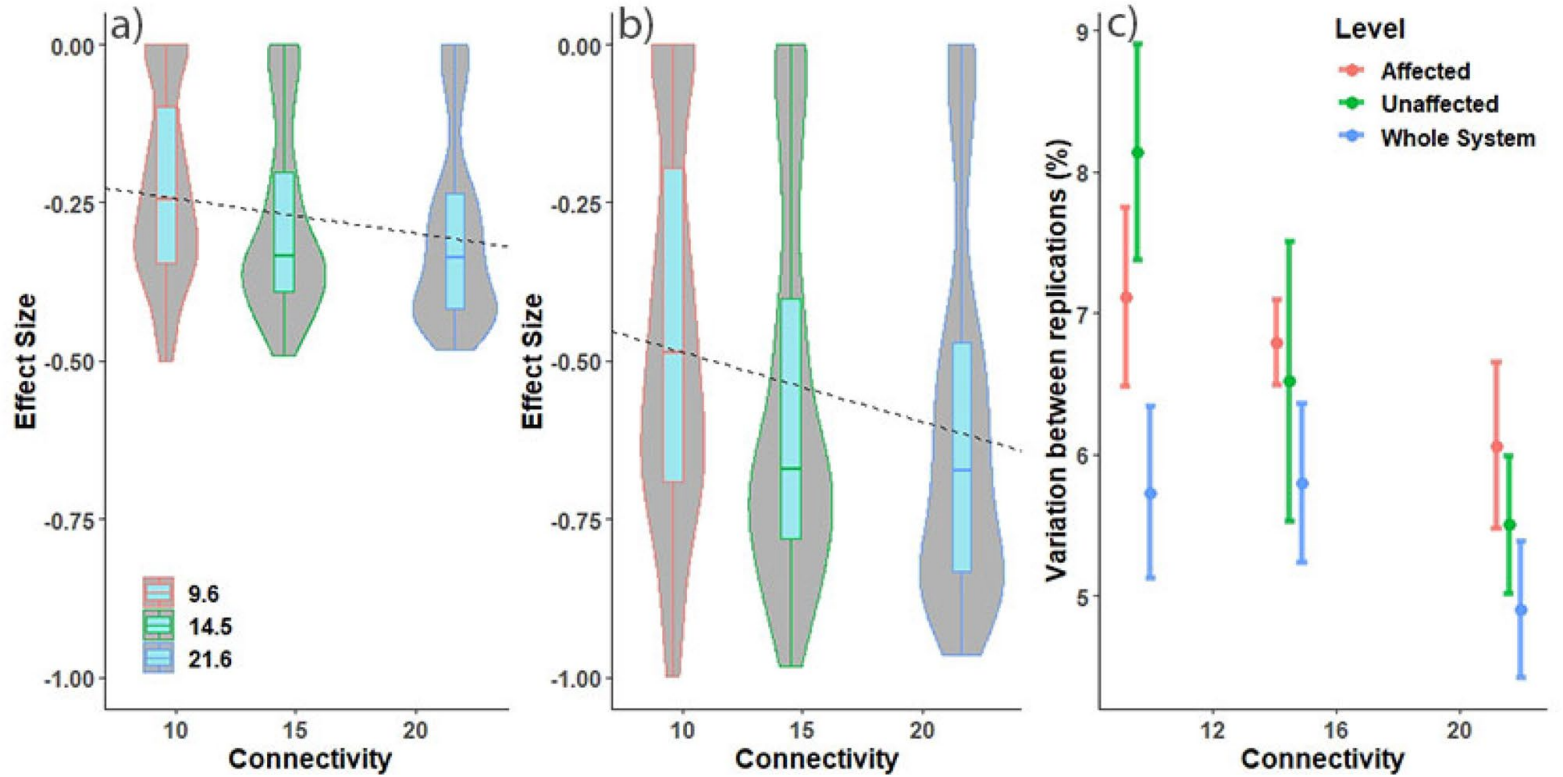
Box and violin plots of effect size for each of the (**a**) 50% and (**b**) 100% disease effect scenarios and (**c**) standard error plot of variation across all spatial scales and connectivity levels. Linear model (dashed line) in (**a**) and (**b**) provided as visual guide of direction of trend. Data in (**c**) are the grand-averages of within-treatment standard errors.

We applied a 50% consumption penalty for infected individuals in disease simulations, representing a realistic simulation of real-world diseases that can hinder animal performance. White-spot disease, for example, affects shrimp consumption rates before causing mortality, allowing the disease to spread while also suppressing consumption rates^27^. Some diseases may have different magnitudes of effect on individuals. Thus, we also simulated stronger disease effects by reducing grazing rates for infected crabs by 100%. In general, the simulated disease infected more crabs at higher connectivity levels (Extended data Fig. 3), leading to higher consumption penalties with increasing connectivity in most scenarios (Fig. 3a,b). The 50% disease effect level did not negate the inherent benefits of connectivity observed in unperturbed systems (Fig. 2), but at a more strongly negative relationship between connectivity and grazing performance was observed under a 100% penalty (Fig. 3b). Thus, disease had a higher impact on ecosystem function in better-connected systems (Fig. 3; Extended data Fig. 4).

There was also less variability in grazing performance with higher connectivity, but not significantly (Fig. 3c). The within-treatment standard error in grazing performance was empirically lowest in the best-connected systems across three spatial assessment levels (Fig. 3c), suggesting that grazing responses were more predictable in better-connected systems (Fig. 3c).

### Conservation implications

Given the setting of reserves in complex spatial mosaics, with multiple stressors, it is necessary to have a better understanding of how connectivity can change the way ecosystems respond to stressors. We show that these relationships are complex, even in simplified, controlled systems. Despite the microcosm scale of our experiments, our results support real-world phenomena that have been linked with the benefits of connectivity and/or protected areas. Thus, we suggest these findings contribute valuable information to support the future design of research and management strategies for natural systems. For example, Marine Protected Areas (MPAs) can be considered analogous refuges to the unaffected patches in our harvesting scenarios. MPAs provide an offsetting service in natural ecosystems where, by excluding harvesting, they provide a refuge and source of animal resupply^28^ that supports fisheries and acts to maintain ecosystem function e.g.^22^. Our systems responded similarly to these real-world examples in that the number of animals available for harvest was highest at the edge of the simulated MPA, as observed in the best-connected systems (Extended data Fig. 5). This phenomenon was strongest in the multiple-stressor scenario that applied heat-stress to the patch that was protected from harvesting pressure. Heat-stressed animals vacated the hotter protected area, exposing them to possible capture.

Additionally, connectivity may provide a stabilising effect on ecosystem function, a phenomena that may partially contribute to previous findings that connectivity strengthens ecosystem resilience (e.g.^29^). Thus, when connectivity is low, ecosystems may experience greater variability in the performance of key ecosystem functions, potentially limiting capacity to resist or recover from disturbance.

We tested the role that connectivity plays in shaping animal functional responses to single and multiple disturbance events of different types. To do so, we quantified the effect that different combinations of stressors had on the grazing performance of a widespread mesograzer, the yellow-footed hermit crab (*Clibanarius virescens*), in purpose-built arenas at three levels of connectivity. Connectivity can be measured in many ways, with effects being difficult to quantify between systems with different numbers of redundant or complimentary routes, motifs such as triangular or circular clusters, or ‘hubs’ that connect multiple patches to one central node^30^. To minimise unintended effects of altering the number or place of connections, we altered system connectivity by varying the location of important patches (containing food) within a standard 4 × 3 node grid of approx. 42 × 32 cm (Fig. 1), rather than by adding or removing connections. We selected system configurations (habitat patch placements) that were symmetrical along both the x and y axes, minimising the risk of introducing unintentional confounding effects. This created a base system with 12 nodes and 14 edges in all cases.

Connectivity within each system was calculated using a modified measure of closeness^31^, as in Eq. (1):

1 $$\text{Connectivity }= \frac{1}{T+P+D}\times 100$$

where; T = average shortest path length from food node 1 to all other nodes; P = average shortest path length from food node 2 to all other nodes; and D = shortest path length between both food nodes. All path lengths were counted as integer steps between nodes.

### Standard experimental procedure for all treatments

We tested ten treatments at each connectivity level, resulting in N = 236 replications; between 73 and 82 at each connectivity level.

Each replication involved slowly warming crabs and arenas to the desired temperature (defined per treatment below) over a 4-h period, approximately mimicking daily warming cycles. One crab was then added to each patch (12 total in the system), and six 1 mg algal pellets were added to each of the two food patches (coloured patches in Fig. 1). Every 20 min, the number of pellets remaining was counted, and an additional six pellets were added to each patch. Each experimental replication lasted 1 h (3 × 20-min intervals), starting when crabs were first added to the arena. Thus, in all cases, 18 pellets were added to each patch; 36 total to the system. This was determined as the control level because an individual crab is expected to consume approximately three pellets per hour at an optimal temperature 29.5 °C^25^. Hence, by adding pellets equal to the mean consumption rate (one per crab per 20-min period), we simulated a stable system in which consumption was approximately equal to algal production in the absence of stressors. Any reduction in consumption (driven by stressors) below optimal rates implies that algal mass would increase over time, suggesting that the system is trending towards a phase shift. Thus, by our definition, lower consumption makes the system less resilient.

### Treatment specifics

#### Control

The control treatments were run as per the standard experimental procedure described above with no stressor applied.

#### Heat-stress

For the temperature stressor treatment, the experiment was run as per the standard experimental procedure, but with a temperature stress applied to the half of the arena incorporating an affected patch ‘Zone B’). Water was heated using a combination of sous vide precision cookers and aquarium heaters, arranged in a way that ensured the stability of target temperatures for the duration of the experiment. The stressed half of the arena was set to 33.5 °C, and the unstressed half was set to optimal temperature (29.5 °C; as per^24^). The 4 °C increase in temperature is expected to decrease consumption rates by approx. 15–20%^24^, with an additional effect on movement expected to amplify this effect. This is intended to simulate a system with connected heat-stressed and refuge areas.

#### Harvesting pressure

A harvesting stressor was applied that simulated a fishery management scenario with a fixed bag limit. We designated one food patch as a protected area and resupply point (blue in Fig. 1), and the other as a harvesting area (red in Fig. 1). Because our systems included two distinct habitat patches, separated by different amounts of featureless habitat at different connectivity levels, we equate these to reef or vegetated habitat where fauna are likely to congregate near resources. Similarly, fishers are likely to congregate in the same areas, unless excluded. Thus, by restricting harvesting in one of the patches, we have simulated the broad dynamics of an enclosed bay that contains both protected and high harvesting pressure habitat areas, and some unprotected, but featureless areas in-between that would be expected to experience low harvesting pressure, which we did not attempt to simulate.

In this scenario, we set a bag-limit that allowed up to three crabs (or as many as were present under three) to be harvested from the affected patch at the end of each 20 min interval, and then the same number of crabs were added to the protected area, simulating a maximum sustainable yield management (MSY) scenario. Under the harvesting only stressor, all other experimental procedures were as per the standard scenario, with temperature set to optimal (29.5 °C) across the entire arena.

#### Heat-stress and harvesting

To investigate how multiple stressors interact with the connectivity-resilience relationship, we also applied both heat-stress and harvesting to the same arenas simultaneously in two ways. First, by applying both stressors to the same patch (same), and second by applying the heat-stress to the protected area and the harvesting pressure to the other (diff).

#### Disease

We applied a simulated effect of disease by recording interactions between individuals and applying a 50% and 100% (separately) consumption penalty for ‘infected’ crabs. Reduced consumption is a known effect observed in individuals infected by numerous diseases, including some that are known to affect crustacean mesograzers (e.g. white-spot disease^27^). We recorded all experimental treatments on video (GoPro models) and then extracted data on the movement and interactions between crabs from each video. Unique colour markers were used to track individual crabs, enabling data to be recorded for all occasions during which contact was made between crabs (including the total duration of each interaction). We also recorded the time that each crab entered and/or exited one of the designated habitat patches.

Disease spread scenarios were then modelled from interaction data, whereby we designated an individual crab as being the infection vector (starting with a disease) and then quantified how the disease spread through the system through crab-to-crab interactions. For each treatment replication, 12 sub-replications were assessed simulating each of the 12 different crabs starting with the disease. See Extended data Fig. 1 for example of infection pathways taken for each ‘starting crab’ during one physical replicate. Interaction and movement data were extracted from videos manually. Disease spread was then simulated from interaction data in R using customised code.

For each disease spread scenario we applied the stressor as a 50% reduction in consumption for diseased crabs as our primary test level, also testing the effect at a 100% reduction level to observe a worst-case scenario. The effect was calculated based on the total consumption within a period and time that crabs (both infected and uninfected) spent in close proximity to food using the below equations:

1. *Consumption after disease* = 
  *Disease effect (as percentage)* × *observed consumption rate* × *cumulative infection time.*
2. *Observed consumption rate* = $\frac{total \; consumption}{\sum_{n=1}^{12} crab \; n \; total \; time \; in \; food \; patch}$
3. *Cumulative infection time* = $\sum_{n=1}^{12}crab \; n \; time \; in \; food \; patch \; while \; infected$

See worked example in Extended data Table 1.

### Statistical analyses

We assessed the effect of treatment (connectivity level and stressor(s)) with generalised linear models (glm). Effect was assessed at two levels: both at the *whole system* level, as total consumption across both habitat patches combined and, within the *affected patch* only (affected), tested in separate analyses. Treatments (as factors) were: Control; heat-stress, harvesting; heat-stress and harvesting (same); heat-stress and harvesting (diff). Noting that in the heat-stress and harvesting (diff) treatment there was no designated ‘unaffected’ patch because both patches were affected by at least one stressor. For consistency, we included the patch affected by harvesting in all affected patch analyses. Connectivity level was the system’s connectivity metric (9.6; 14.5; 21.6), also set as a factor.

To identify the best model, we started with the most complex model that included all possible interaction terms, and used a leave-one-out technique, exclude the most complex interaction term until a significant interaction was identified using Analysis of Deviance (ANOVA) with chi-squared test (detailed outputs in S1; S2). The final glm model was selected as the most complex equation (i.e. largest interaction term) that returned a significant interaction in this test, resulting in a final model for both system level and affected patch level of:

*Consumption ~ Harvesting* + *Heat-stress* + *Connectivity* + *Disease* + *Harvesting:heat-stress* + *Harvesting:Connectivity* + *Heat-stress:Connectivity* + *Harvesting:Disease* + *Heat-stress:Disease* + *Connectivity:Disease* + *Harvesting:Heat-stress:Connectivity.*

Mean ± SE plots presented were derived from outputs for this glm equation. Alpha was set to 0.05.

Treatment differences were assessed using model outputs, with significant differences defined as non-overlapping treatment values for model fit (mean) ± standard error.

Disease effect size was calculated as:

$$Effect\;size \, = \, \left( {diseased\;consumption \, {-} \, replication\;consumption} \right)/replication\;consumption$$

## Conclusions

Key ecosystem functions in unstressed systems were positively linked to connectivity in our experimental microcosms. While stressors reduced function across all connectivity levels, the benefits of better connectivity were maintained under spatially explicit stressors (e.g. heat-stress, animal harvesting), but reversed in disease scenarios where morbid individuals interact more frequently in better-connected systems. Such contrasting effects of connectivity may present a challenge for conservation and management if trends are consistent with those in more complex natural systems. Our experimental technique and findings provide a platform for future studies to better understand how connectivity affects resilience in more complex systems and at larger spatial scales.

## Supplementary Information

Supplementary Information 1.

Supplementary Information 2.
