# Supplementary material for: Disturbance type determines how connectivity shapes ecosystem resilience
Source: Sci Rep. 2021 Jan 13;11:1188. doi: 10.1038/s41598-021-80987-1 (PMC7806881; doi:10.1038/s41598-021-80987-1)
Supplement: Supplementary file 1 — Supplementary Information 1. [file 41598_2021_80987_MOESM1_ESM.docx]

**Disturbance type determines how connectivity shapes ecosystem resilience**

Ryan M. Pearson*^1^, Thomas A. Schlacher^2^, Kristin I. Jinks^1^, Andrew D. Olds^2^, Christopher J. Brown^1^, Rod M. Connolly^1^

**Disturbance type determines how connectivity shapes ecosystem resilience**

**Extended data**


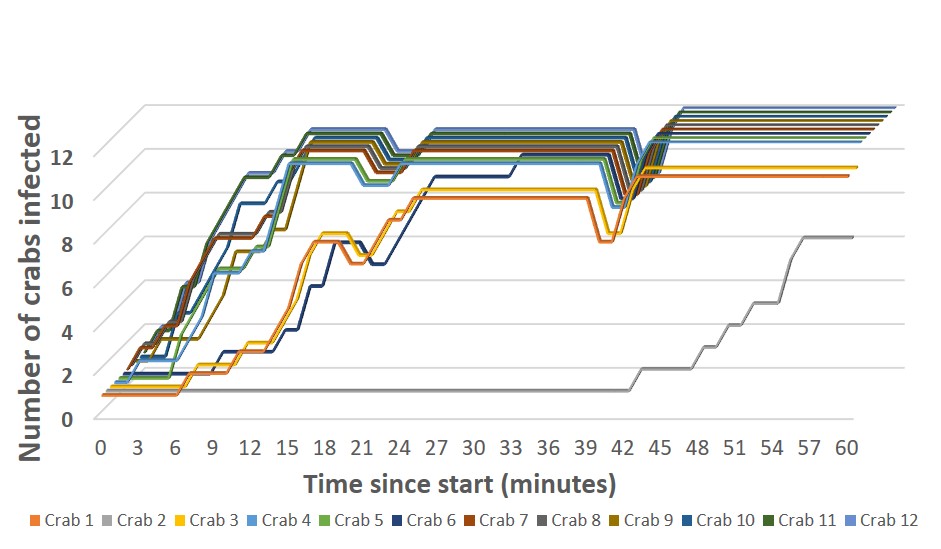


***Extended Data* Fig *1*.** Example of the cumulative number of crabs infected per minute within a single replication. Each line represents the disease spread scenario when a different individual crab starts with the disease. This example treatment included harvesting as a stressor, thus the number of infected crabs sometimes decreases at 20 and 40 minutes when some infected crabs were removed from the system at these points.

**Extended Data Table 1.** Example of infection pathway and overall effect of digital disease on consumption. This represents the effect of one crab starting with the disease (#7) during a single assay. This treatment included harvesting pressure, hence a total of 18 crabs were involved (6 removed, and 6 resupplied, leaving 12 in the system at all times)

| **Crab number** | **Infection time** | | **Time spent in zone after infection** | | |
| --- | --- | --- | --- | --- | --- |
|  |  |  | **0-20 mins** | **20-40 mins** | **40-60 mins** |
| 7 | 0:00:00 | |  |  |  |
| 10 | 0:00:17 | |  |  |  |
| 11 | 0:00:47 | |  |  |  |
| 6 | 0:01:03 | | 0:08:36 | 0:20:00 | 0:20:00 |
| 8 | 0:01:03 | |  |  |  |
| 2 | 0:05:19 | | 0:04:15 |  |  |
| 3 | 0:08:27 | | 0:11:33 | 0:20:00 | 0:20:00 |
| 5 | 0:14:28 | | 0:05:32 | 0:20:00 | 0:20:00 |
| 4 | 0:16:51 | | 0:03:09 | 0:04:33 |  |
| 9 | 0:19:07 | |  |  |  |
| 13 | 0:20:59 | |  | 0:00:45 |  |
| 15 | 0:21:24 | |  | 0:18:36 | 0:19:28 |
| 1 | 0:30:04 | |  |  |  |
| 12 | 0:39:03 | |  |  |  |
| 16 | 0:41:58 | |  |  | 0:18:02 |
| 18 | 0:55:39 | |  |  | 0:04:21 |
| 14 | 0:58:14 | |  |  | 0:01:46 |
| 17 | N/A | |  |  |  |
| **Total pellets consumed (observed)** | | | 5 | 6 | 6 |
| **Crab-minutes (cumulative time-in-zone for all crabs)** | | | 71.23 | 106.28 | 149.30 |
| **Pellets consumed per crab-minute** | | | 0.070 | 0.057 | 0.040 |
| **Infected crab-minutes (cum. time-in-zone for infected crabs)** | | | 33.08 | 83.90 | 103.62 |
| **Pellets consumed while infected** | | | 2.32 | 4.78 | 4.14 |
| **50% reduction on disease consumption** | | | 1.16 | 2.39 | 2.07 |
| **Pellets while not infected** | | | 2.68 | 1.21 | 1.86 |
| **Total consumption with 50% effect** | |  | **3.84** | **3.61** | **3.93** |
| **Disease effect (reduction in total consumption)** | |  | **-23%** | **-40%** | **-35%** |

**
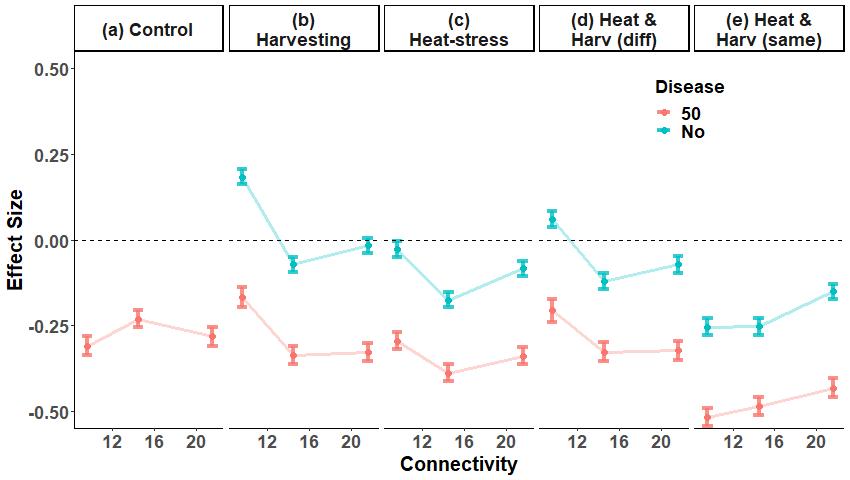
**

**Extended data Fig. 2.** Proportional change in algal consumption between control and each treatment. X-axis represents connectivity level. Colours represent non-disease replications (No) and the same replications with 50% disease penalty applied (50).


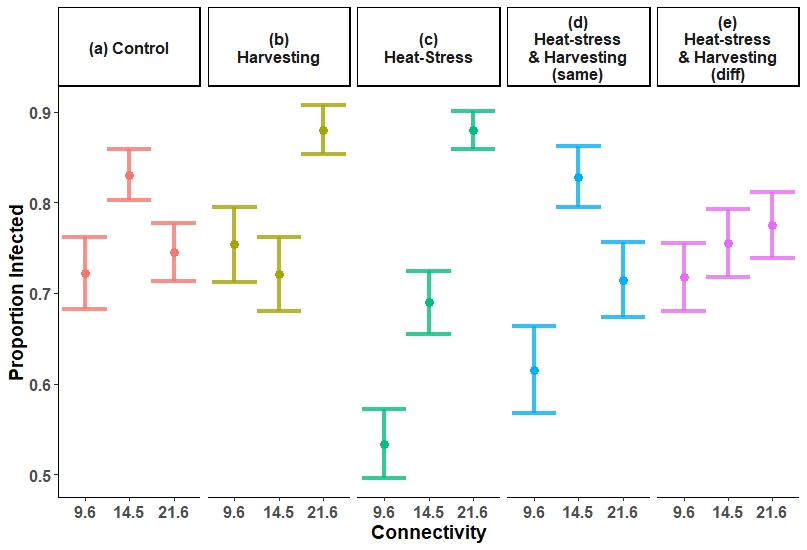


**Extended data Fig 3.** Proportion of total animals that were infected after 60 minutes (mean ± SE). **a & c** had 12 animals in all cases (no harvesting pressure). **b, d & e:** had between 12 & 18 animals, dependent on the number of animals available for harvest at each interval.


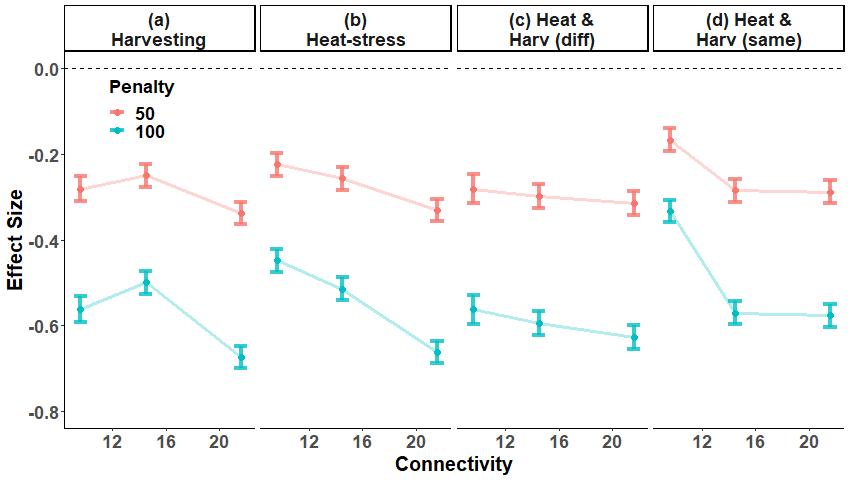


**Extended data Fig 4.** System level disease effect per treatment. Calculated as proportional change in algal consumption from treatment consumption, after a disease effect (50, 100) is added to each treatment.

**Extended data Fig. 5.** Animals harvested during treatments that included a harvesting pressure at each connectivity level. Mean ± SE as percentage of total allowed by designated bag-limit (max 3 per interval, if available).
