# Supplementary material for: Disturbance type determines how connectivity shapes ecosystem resilience
Source: Sci Rep. 2021 Jan 13;11:1188. doi: 10.1038/s41598-021-80987-1 (PMC7806881; doi:10.1038/s41598-021-80987-1)
Supplement: Supplementary file 2 — Supplementary Information 2. [file 41598_2021_80987_MOESM2_ESM.docx]

**Disturbance type determines how connectivity shapes ecosystem resilience**

Ryan M. Pearson*^1^, Thomas A. Schlacher^2^, Kristin I. Jinks^1^, Andrew D. Olds^2^, Christopher J. Brown^1^, Rod M. Connolly^1^

**Supplementary – Supporting results**

S1: System level - 50% disease analyses

Analysis of Deviance Table

Model 1: cbind(System, system_not_eaten) ~ Harvesting + tempf + Connectivityf + Disease + Harvesting:tempf + Harvesting:Connectivityf + tempf:Connectivityf + Harvesting:Disease + tempf:Disease + Connectivityf:Disease + Harvesting:tempf:Connectivityf

Model 2: cbind(System, system_not_eaten) ~ Harvesting + tempf + Connectivityf + Disease + Harvesting:tempf + Harvesting:Connectivityf + tempf:Connectivityf + Harvesting:Disease + tempf:Disease + Connectivityf:Disease

|  | **Resid. Df** | **Resid. Dev** | **Df** | **Deviance** | **Pr (>Chi)** |
| --- | --- | --- | --- | --- | --- |
| **1** | 215 | 864.03 |  |  |  |
| **2** | 217 | 877.49 | -2 | -13.462 | 0.001 ** |

Signif. codes: 0 ‘***’ 0.001 ‘**’ 0.01 ‘*’ 0.05 ‘.’ 0.1 ‘ ’ 1

**System level glm model outputs with significant three-way interaction**

__________________________________________________________________________

Call:

glm(formula = cbind(System, system_not_eaten) ~ Harvesting +

tempf + Connectivityf + Disease + Harvesting:tempf + Harvesting:Connectivityf +

tempf:Connectivityf + Harvesting:Disease + tempf:Disease +

Connectivityf:Disease + Harvesting:tempf:Connectivityf, family = "binomial",

data = dat50s)

Deviance Residuals:

Min 1Q Median 3Q Max

-5.5313 -1.1378 -0.0263 1.2258 4.9077

Coefficients: (3 not defined because of singularities)

|  | **Estimate** | **Std. Error** | **z value** | **p** | **sig.** |
| --- | --- | --- | --- | --- | --- |
| (Intercept) | 0.01897 | 0.09196 | 0.206 | 0.836542 |  |
| HarvestingYes | 0.37525 | 0.12659 | 2.964 | 0.003032 | ** |
| tempfA | -0.2523 | 0.12973 | -1.945 | 0.051805 | . |
| tempfB | -0.05325 | 0.12601 | -0.423 | 0.672613 |  |
| Connectivityf14.5 | 0.78707 | 0.12736 | 6.18 | 6.41E-10 | *** |
| Connectivityf21.6 | 0.65347 | 0.12921 | 5.058 | 4.25E-07 | *** |
| DiseaseYes | -0.64346 | 0.11833 | -5.438 | 5.40E-08 | *** |
| HarvestingYes:tempfA | NA | NA | NA | NA |  |
| HarvestingYes:tempfB | -0.84414 | 0.17992 | -4.692 | 2.71E-06 | *** |
| HarvestingYes:Connectivityf14.5 | -0.5974 | 0.17222 | -3.469 | 0.000523 | *** |
| HarvestingYes:Connectivityf21.6 | -0.42372 | 0.17301 | -2.449 | 0.014321 | * |
| tempfA:Connectivityf14.5 | 0.10725 | 0.17529 | 0.612 | 0.540646 |  |
| tempfB:Connectivityf14.5 | -0.47115 | 0.16893 | -2.789 | 0.005286 | ** |
| tempfA:Connectivityf21.6 | 0.09458 | 0.17624 | 0.537 | 0.591496 |  |
| tempfB:Connectivityf21.6 | -0.1839 | 0.16979 | -1.083 | 0.278772 |  |
| HarvestingYes:DiseaseYes | -0.071 | 0.10586 | -0.671 | 0.502401 |  |
| tempfA:DiseaseYes | 0.16987 | 0.14107 | 1.204 | 0.228531 |  |
| tempfB:DiseaseYes | 0.08675 | 0.10546 | 0.823 | 0.410773 |  |
| Connectivityf14.5:DiseaseYes | -0.0329 | 0.12071 | -0.273 | 0.785201 |  |
| Connectivityf21.6:DiseaseYes | -0.12792 | 0.12028 | -1.063 | 0.287556 |  |
| HarvestingYes:tempfA:Connectivityf14.5 | NA | NA | NA | NA |  |
| HarvestingYes:tempfB:Connectivityf14.5 | 0.85352 | 0.24773 | 3.445 | 0.00057 | *** |
| HarvestingYes:tempfA:Connectivityf21.6 | NA | NA | NA | NA |  |
| HarvestingYes:tempfB:Connectivityf21.6 | 0.70843 | 0.24575 | 2.883 | 0.003942 | ** |

---

Signif. codes: 0 ‘***’ 0.001 ‘**’ 0.01 ‘*’ 0.05 ‘.’ 0.1 ‘ ’ 1

(Dispersion parameter for binomial family taken to be 1)

Null deviance: 1225.23 on 235 degrees of freedom

Residual deviance: 864.03 on 215 degrees of freedom

AIC: 1819

Number of Fisher Scoring iterations: 4

**Table S1. Whole system:** Model estimates from glm tested effect of physical treatments (heat-stress & harvesting) and 50% simulated disease effect on consumption across the **whole system** at three connectivity levels. Within-treatment groups defined by connectivity levels within each of the 10 treatments that have overlapping SE. Treatment vs Control significance derived from comparison of treatment SE range to Control SE range at same connectivity level. Non-overlap = significant difference.

| **Treatment** | **Connectivity** | **Patch affected by heat-stress** | **Patch affected by Harvesting** | **Fit (mean)** | **CI** | **fit-CI** | **fit+CI** | **SE** | **fit-SE** | **fit+SE** | **Within treatment groups** | **Treatment vs Control** |
| --- | --- | --- | --- | --- | --- | --- | --- | --- | --- | --- | --- | --- |
| **Control** | 9.6 | none | none | **0.50** | 0.05 | 0.46 | 0.55 | 0.02 | 0.48 | 0.53 | A | N/A |
| **Control** | 14.5 | none | none | **0.69** | 0.04 | 0.65 | 0.73 | 0.02 | 0.67 | 0.71 | B | N/A |
| **Control** | 21.6 | none | none | **0.66** | 0.04 | 0.62 | 0.70 | 0.02 | 0.64 | 0.68 | B | N/A |
| **Harvesting** | 9.6 | none | B | **0.60** | 0.04 | 0.55 | 0.64 | 0.02 | 0.57 | 0.62 | A | ****sig. higher** |
| **Harvesting** | 14.5 | none | B | **0.64** | 0.04 | 0.60 | 0.69 | 0.02 | 0.62 | 0.66 | AB | ****sig. lower** |
| **Harvesting** | 21.6 | none | B | **0.65** | 0.04 | 0.61 | 0.69 | 0.02 | 0.63 | 0.67 | B | Ns |
| **Heat-stress** | 9.6 | B | none | **0.49** | 0.05 | 0.45 | 0.54 | 0.02 | 0.47 | 0.51 | A | Ns |
| **Heat-stress** | 14.5 | B | none | **0.57** | 0.04 | 0.53 | 0.61 | 0.02 | 0.55 | 0.59 | AB | ****sig. lower** |
| **Heat-stress** | 21.6 | B | none | **0.61** | 0.04 | 0.56 | 0.65 | 0.02 | 0.59 | 0.63 | B | ****sig. lower** |
| **Heat & Harv (diff)** | 9.6 | A | B | **0.54** | 0.05 | 0.49 | 0.58 | 0.02 | 0.51 | 0.56 | A | Ns |
| **Heat & Harv (diff)** | 14.5 | A | B | **0.61** | 0.05 | 0.56 | 0.65 | 0.02 | 0.58 | 0.63 | B | ****sig. lower** |
| **Heat & Harv (diff)** | 21.6 | A | B | **0.61** | 0.05 | 0.57 | 0.66 | 0.02 | 0.59 | 0.64 | B | ****sig. lower** |
| **Heat & Harv (same)** | 9.6 | B | B | **0.38** | 0.05 | 0.33 | 0.42 | 0.02 | 0.35 | 0.40 | A | ****sig. lower** |
| **Heat & Harv (same)** | 14.5 | B | B | **0.52** | 0.05 | 0.47 | 0.57 | 0.02 | 0.49 | 0.54 | B | ****sig. lower** |
| **Heat & Harv (same)** | 21.6 | B | B | **0.56** | 0.04 | 0.52 | 0.61 | 0.02 | 0.54 | 0.58 | B | ****sig. lower** |
| **Control + disease** | 9.6 | none | none | **0.35** | 0.06 | 0.29 | 0.40 | 0.03 | 0.32 | 0.38 | A | ****sig. lower** |
| **Control + disease** | 14.5 | none | none | **0.53** | 0.05 | 0.48 | 0.58 | 0.03 | 0.51 | 0.56 | B | ****sig. lower** |
| **Control + disease** | 21.6 | none | none | **0.48** | 0.05 | 0.42 | 0.53 | 0.03 | 0.45 | 0.50 | C | ****sig. lower** |
| **Harvesting + disease** | 9.6 | none | B | **0.42** | 0.06 | 0.36 | 0.48 | 0.03 | 0.39 | 0.45 | A | ****sig. lower** |
| **Harvesting + disease** | 14.5 | none | B | **0.46** | 0.05 | 0.41 | 0.51 | 0.03 | 0.43 | 0.49 | A | ****sig. lower** |
| **Harvesting + disease** | 21.6 | none | B | **0.45** | 0.05 | 0.40 | 0.50 | 0.03 | 0.42 | 0.47 | A | ****sig. lower** |
| **Heat-stress + disease** | 9.6 | B | none | **0.36** | 0.05 | 0.31 | 0.41 | 0.03 | 0.33 | 0.38 | A | ****sig. lower** |
| **Heat-stress + disease** | 14.5 | B | none | **0.42** | 0.05 | 0.37 | 0.48 | 0.03 | 0.40 | 0.45 | B | ****sig. lower** |
| **Heat-stress + disease** | 21.6 | B | none | **0.44** | 0.05 | 0.39 | 0.49 | 0.03 | 0.41 | 0.46 | B | ****sig. lower** |
| **Heat & Harv (diff) + disease** | 9.6 | A | B | **0.40** | 0.07 | 0.33 | 0.47 | 0.03 | 0.37 | 0.43 | A | ****sig. lower** |
| **Heat & Harv (diff) + disease** | 14.5 | A | B | **0.47** | 0.06 | 0.41 | 0.52 | 0.03 | 0.44 | 0.49 | B | ****sig. lower** |
| **Heat & Harv (diff) + disease** | 21.6 | A | B | **0.45** | 0.06 | 0.39 | 0.50 | 0.03 | 0.42 | 0.48 | AB | ****sig. lower** |
| **Heat & Harv (same) + disease** | 9.6 | B | B | **0.24** | 0.05 | 0.19 | 0.30 | 0.03 | 0.22 | 0.27 | A | ****sig. lower** |
| **Heat & Harv (same) + disease** | 14.5 | B | B | **0.36** | 0.05 | 0.30 | 0.41 | 0.03 | 0.33 | 0.38 | B | ****sig. lower** |
| **Heat & Harv (same) + disease** | 21.6 | B | Yes | **0.38** | 0.05 | 0.32 | 0.43 | 0.03 | 0.35 | 0.40 | B | ****sig. lower** |

S2: Affected patch – 50%

**Analysis of Deviance Table**

**Model 1:** cbind(ZoneB, zoneB_not_eaten) ~ Harvesting + tempf + Connectivityf + Disease + Harvesting:tempf + Harvesting:Connectivityf + tempf:Connectivityf + Harvesting:Disease + tempf:Disease + Connectivityf:Disease +

Harvesting:tempf:Connectivityf

**Model 2:** cbind(ZoneB, zoneB_not_eaten) ~ Harvesting + tempf + Connectivityf + Disease + Harvesting:tempf + Harvesting:Connectivityf + tempf:Connectivityf + Harvesting:Disease + tempf:Disease + Connectivityf:Disease

|  | **Resid. Df** | **Resid. Dev** | **Df** | **Deviance** | **Pr (>Chi)** |
| --- | --- | --- | --- | --- | --- |
| **1** | 215 | 693.93 |  |  |  |
| **2** | 217 | 703.37 | -2 | -9.44 | 0.009 ** |

---

Signif. codes: 0 ‘***’ 0.001 ‘**’ 0.01 ‘*’ 0.05 ‘.’ 0.1 ‘ ’ 1

Call:

glm(formula = cbind(ZoneB, zoneB_not_eaten) ~ Harvesting + tempf + Connectivityf + Disease + Harvesting:tempf + Harvesting:Connectivityf + tempf:Connectivityf + Harvesting:Disease + tempf:Disease + Connectivityf:Disease + Harvesting:tempf:Connectivityf, family = "binomial", data = dat50.2)

Deviance Residuals: Min 1Q Median 3Q Max

-4.8632 -1.1530 -0.0378 1.0684 6.0792

Coefficients: (3 not defined because of singularities) |

Signif. codes: 0 ‘***’ 0.001 ‘**’ 0.01 ‘*’ 0.05 ‘.’ 0.1 ‘ ’ 1

|  | Estimate | Std. Error | z value | Pr(>\|z\|) | Sig. code |
| --- | --- | --- | --- | --- | --- |
| (Intercept) | -0.11971 | 0.1312 | -0.912 | 0.36156 |  |
| HarvestingYes | -0.28168 | 0.18198 | -1.548 | 0.12166 |  |
| tempfA | 0.80954 | 0.18648 | 4.341 | 1.42E-05 | *** |
| tempfB | -0.46336 | 0.18412 | -2.517 | 0.01185 | * |
| Connectivityf14.5 | 1.04751 | 0.18352 | 5.708 | 1.14E-08 | *** |
| Connectivityf21.6 | 0.76858 | 0.18399 | 4.177 | 2.95E-05 | *** |
| DiseaseYes | -0.71978 | 0.17567 | -4.097 | 4.18E-05 | *** |
| HarvestingYes:tempfA | NA | NA | NA | NA |  |
| HarvestingYes:tempfB | -0.74009 | 0.28518 | -2.595 | 0.00945 | ** |
| HarvestingYes:Connectivityf14.5 | -0.7245 | 0.24714 | -2.932 | 0.00337 | ** |
| HarvestingYes:Connectivityf21.6 | -0.3463 | 0.24687 | -1.403 | 0.16069 |  |
| tempfA:Connectivityf14.5 | -0.78718 | 0.2495 | -3.155 | 0.0016 | ** |
| tempfB:Connectivityf14.5 | -0.48564 | 0.24468 | -1.985 | 0.04717 | * |
| tempfA:Connectivityf21.6 | -0.46619 | 0.25087 | -1.858 | 0.06313 | . |
| tempfB:Connectivityf21.6 | -0.07676 | 0.24432 | -0.314 | 0.75338 |  |
| HarvestingYes:DiseaseYes | 0.07418 | 0.15827 | 0.469 | 0.6393 |  |
| tempfA:DiseaseYes | -0.01035 | 0.20371 | -0.051 | 0.95949 |  |
| tempfB:DiseaseYes | 0.20517 | 0.15699 | 1.307 | 0.19123 |  |
| Connectivityf14.5:DiseaseYes | 0.08962 | 0.1805 | 0.497 | 0.61951 |  |
| Connectivityf21.6:DiseaseYes | -0.05207 | 0.18004 | -0.289 | 0.77242 |  |
| HarvestingYes:tempfA:Connectivityf14.5 | NA | NA | NA | NA |  |
| HarvestingYes:tempfB:Connectivityf14.5 | 1.13485 | 0.37614 | 3.017 | 0.00255 | ** |
| HarvestingYes:tempfA:Connectivityf21.6 | NA | NA | NA | NA |  |
| HarvestingYes:tempfB:Connectivityf21.6 | 0.49425 | 0.37369 | 1.323 | 0.18595 |  |

**Table S2. Affected patch:** Model estimates from glm tested effect of physical treatments (heat-stress & harvesting) and 50% simulated disease effect on consumption within the **affected patch** across three connectivity levels. Estimates for patch ‘B’ in all cases. Within-treatment groups defined by connectivity levels within each of the 10 treatments that have overlapping SE. Treatment vs Control significance derived from comparison of treatment SE range to Control SE range at same connectivity level. Non-overlap = significant difference.

| **Treatment** | **Connectivity** | **Patch affected by heat-stress** | **Patch affected by harvesting** | **fit** | **CI** | **fit-CI** | **fit+CI** | **SE** | **fit-SE** | **fit+SE** | **Within treatment groups** | **Treatment vs Control** |
| --- | --- | --- | --- | --- | --- | --- | --- | --- | --- | --- | --- | --- |
| **Control** | 9.6 | none | none | **0.50** | 0.05 | 0.46 | 0.55 | 0.02 | 0.48 | 0.53 | A | N/A |
| **Control** | 14.5 | none | none | **0.69** | 0.04 | 0.65 | 0.73 | 0.02 | 0.67 | 0.71 | B | N/A |
| **Control** | 21.6 | none | none | **0.66** | 0.04 | 0.62 | 0.70 | 0.02 | 0.64 | 0.68 | B | N/A |
| **Harvesting** | 9.6 | none | B | **0.60** | 0.04 | 0.55 | 0.64 | 0.02 | 0.57 | 0.62 | A | ****sig. higher** |
| **Harvesting** | 14.5 | none | B | **0.64** | 0.04 | 0.60 | 0.69 | 0.02 | 0.62 | 0.66 | AB | ****sig. lower** |
| **Harvesting** | 21.6 | none | B | **0.65** | 0.04 | 0.61 | 0.69 | 0.02 | 0.63 | 0.67 | B | ns |
| **Heat-stress** | 9.6 | B | none | **0.49** | 0.05 | 0.45 | 0.54 | 0.02 | 0.47 | 0.51 | A | ns |
| **Heat-stress** | 14.5 | B | none | **0.57** | 0.04 | 0.53 | 0.61 | 0.02 | 0.55 | 0.59 | B | ****sig. lower** |
| **Heat-stress** | 21.6 | B | none | **0.61** | 0.04 | 0.56 | 0.65 | 0.02 | 0.59 | 0.63 | B | ****sig. lower** |
| **Heat & Harv (diff)** | 9.6 | A | B | **0.54** | 0.05 | 0.49 | 0.58 | 0.02 | 0.51 | 0.56 | A | ns |
| **Heat & Harv (diff)** | 14.5 | A | B | **0.61** | 0.05 | 0.56 | 0.65 | 0.02 | 0.58 | 0.63 | B | ****sig. lower** |
| **Heat & Harv (diff)** | 21.6 | A | B | **0.61** | 0.05 | 0.57 | 0.66 | 0.02 | 0.59 | 0.64 | B | ****sig. lower** |
| **Heat & Harv (same)** | 9.6 | B | B | **0.38** | 0.05 | 0.33 | 0.42 | 0.02 | 0.35 | 0.40 | A | ****sig. lower** |
| **Heat & Harv (same)** | 14.5 | B | B | **0.52** | 0.05 | 0.47 | 0.57 | 0.02 | 0.49 | 0.54 | B | ****sig. lower** |
| **Heat & Harv (same)** | 21.6 | B | B | **0.56** | 0.04 | 0.52 | 0.61 | 0.02 | 0.54 | 0.58 | B | ****sig. lower** |
| **Control + disease** | 9.6 | none | none | **0.35** | 0.06 | 0.29 | 0.40 | 0.03 | 0.32 | 0.38 | A | ****sig. lower** |
| **Control + disease** | 14.5 | none | none | **0.53** | 0.05 | 0.48 | 0.58 | 0.03 | 0.51 | 0.56 | B | ****sig. lower** |
| **Control + disease** | 21.6 | none | none | **0.48** | 0.05 | 0.42 | 0.53 | 0.03 | 0.45 | 0.50 | C | ****sig. lower** |
| **Harvesting + disease** | 9.6 | none | B | **0.42** | 0.06 | 0.36 | 0.48 | 0.03 | 0.39 | 0.45 | A | ****sig. lower** |
| **Harvesting + disease** | 14.5 | none | B | **0.46** | 0.05 | 0.41 | 0.51 | 0.03 | 0.43 | 0.49 | A | ****sig. lower** |
| **Harvesting + disease** | 21.6 | none | B | **0.45** | 0.05 | 0.40 | 0.50 | 0.03 | 0.42 | 0.47 | A | ****sig. lower** |
| **Heat-stress + disease** | 9.6 | B | none | **0.36** | 0.05 | 0.31 | 0.41 | 0.03 | 0.33 | 0.38 | A | ****sig. lower** |
| **Heat-stress + disease** | 14.5 | B | none | **0.42** | 0.05 | 0.37 | 0.48 | 0.03 | 0.40 | 0.45 | B | ****sig. lower** |
| **Heat-stress + disease** | 21.6 | B | none | **0.44** | 0.05 | 0.39 | 0.49 | 0.03 | 0.41 | 0.46 | B | ****sig. lower** |
| **Heat & Harv (diff) + disease** | 9.6 | A | B | **0.40** | 0.07 | 0.33 | 0.47 | 0.03 | 0.37 | 0.43 | A | ****sig. lower** |
| **Heat & Harv (diff) + disease** | 14.5 | A | B | **0.47** | 0.06 | 0.41 | 0.52 | 0.03 | 0.44 | 0.49 | B | ****sig. lower** |
| **Heat & Harv (diff) + disease** | 21.6 | A | B | **0.45** | 0.06 | 0.39 | 0.50 | 0.03 | 0.42 | 0.48 | AB | ****sig. lower** |
| **Heat & Harv (same) + disease** | 9.6 | B | B | **0.24** | 0.05 | 0.19 | 0.30 | 0.03 | 0.22 | 0.27 | A | ****sig. lower** |
| **Heat & Harv (same) + disease** | 14.5 | B | B | **0.36** | 0.05 | 0.30 | 0.41 | 0.03 | 0.33 | 0.38 | B | ****sig. lower** |
| **Heat & Harv (same) + disease** | 21.6 | B | Yes | **0.38** | 0.05 | 0.32 | 0.43 | 0.03 | 0.35 | 0.40 | B | ****sig. lower** |

S3 Simulated disease propagation

**Table S3. Proportion of animals infected with simulated disease after 60 minutes under each stressor-connectivity scenario.**

| **Treatment** | **Connectivity** | **Proportion infected (mean)** | **se** |
| --- | --- | --- | --- |
| **(a) Control** | 9.6 | 0.72 | 0.04 |
| **(a) Control** | 14.5 | 0.83 | 0.03 |
| **(a) Control** | 21.6 | 0.75 | 0.03 |
| **(b) Harvesting** | 9.6 | 0.75 | 0.04 |
| **(b) Harvesting** | 14.5 | 0.72 | 0.04 |
| **(b) Harvesting** | 21.6 | 0.88 | 0.03 |
| **(c) Heat-Stress** | 9.6 | 0.53 | 0.04 |
| **(c) Heat-Stress** | 14.5 | 0.69 | 0.03 |
| **(c) Heat-Stress** | 21.6 | 0.88 | 0.02 |
| **(d) Heat-stress & Harvesting (same)** | 9.6 | 0.62 | 0.05 |
| **(d) Heat-stress & Harvesting (same)** | 14.5 | 0.83 | 0.03 |
| **(d) Heat-stress & Harvesting (same)** | 21.6 | 0.71 | 0.04 |
| **(e) Heat-stress & Harvesting (diff)** | 9.6 | 0.72 | 0.04 |
| **(e) Heat-stress & Harvesting (diff)** | 14.5 | 0.76 | 0.04 |
| **(e) Heat-stress & Harvesting (diff)** | 21.6 | 0.78 | 0.04 |
